# Comprehensive Complication Index Versus Clavien–Dindo Classification After CRS-HIPEC for Colorectal Peritoneal Metastases: Associations with 30- and 90-Day Readmission

**DOI:** 10.3390/medicina62101935

**Published:** 2026-10-07

**Authors:** Uğur Topal, İbrahim Çoğal, Yunus Kaycı, İshak Aydın, Serdar Gümüş, Orçun Yalav, İsmail Cem Eray, Cem Kaan Parsak

**Affiliations:** 1 Department of General Surgery, Division of Surgical Oncology, Faculty of Medicine, Çukurova University, 01330 Adana, Türkiye; cogal.ibrahim@gmail.com (İ.Ç.); seredargumus@hotmail.com (S.G.); ckpadan@gmail.com (C.K.P.); 2 Division of Gastroenterological Surgery, Adana City Training and Research Hospital, 01230 Adana, Türkiye; ykayci@hotmail.com; 3 Department of General Surgery, Faculty of Medicine, Çukurova University, 01330 Adana, Türkiye; drishakaydin@gmail.com (İ.A.); drorcun22@yahoo.com (O.Y.); ismailcemeraygm7@gmail.com (İ.C.E.)

**Keywords:** cytoreductive surgery, hyperthermic intraperitoneal chemotherapy, comprehensive complication index, Clavien–Dindo classification, colorectal cancer, peritoneal metastasis

## Abstract

*Background and Objectives:* To compare the Comprehensive Complication Index (CCI) with the conventional Clavien–Dindo (CD) classification for association with readmission after cytoreductive surgery with hyperthermic intraperitoneal chemotherapy (CRS-HIPEC) for colorectal peritoneal metastases. *Materials and Methods:* Single-center retrospective cohort of 26 patients treated with CRS-HIPEC for peritoneal metastases of colonic or rectal origin. The patient-level CD grade was the highest of the individually graded complications of the index admission; the CCI was calculated with the standard formula. A prespecified threshold of CCI ≥ 26.2—the value of a single CD grade IIIa complication—defined a high cumulative burden. Unplanned readmission was assessed at 30 and 90 days after discharge as co-primary outcomes; classification agreement and correlates of CCI level were exploratory, without adjustment for multiplicity. *Results:* Agreement was moderate (kappa = 0.54, 95% confidence interval [CI] 0.25–0.83). Six of 26 patients (23.1%) had no major complication by the CD classification yet a high cumulative burden by the CCI (exact McNemar *p* = 0.031). Readmission occurred in 32.0% by 30 days and 44.0% by 90 days. For 90-day readmission the CCI gave a numerically higher area under the curve than the CD classification (0.672 vs. 0.555; DeLong *p* = 0.074), and readmission was more frequent above the CCI threshold (55.6% vs. 14.3%; odds ratio 6.95, 95% CI 0.64–378.69); neither comparison was significant. CCI level correlated with body mass index (rho = 0.541) and the Peritoneal Cancer Index (rho = 0.423), both *p* < 0.05. *Conclusions:* The CCI identified a high cumulative burden in six patients recorded as having no major complication by the CD classification, but its numerically higher discrimination for readmission was not statistically demonstrated. These exploratory findings require confirmation in larger cohorts.

## 1. Introduction

Peritoneal metastasis represents one of the most challenging patterns of dissemination in colorectal cancer and has historically been associated with a poor prognosis when treated with systemic therapy alone [1]. In the landmark randomized trial by Verwaal et al., CRS-HIPEC improved median overall survival from 12.6 to 22.3 months compared with the then-standard systemic chemotherapy with or without palliative surgery (*p* = 0.032) [2]. This result arose in an earlier therapeutic era, before current multidrug and biologic systemic regimens, and the trial compared a multimodal CRS-HIPEC strategy rather than isolating the individual effects of surgery and HIPEC. Within the surgical arm, survival was strongly related to completeness of cytoreduction, emphasizing the central contribution of complete tumor removal.

The incremental contribution of HIPEC after complete cytoreductive surgery was re-evaluated in the PRODIGE 7 trial. Adding short-duration oxaliplatin-based HIPEC to CRS did not improve overall survival compared with CRS alone (median, 41.7 vs. 41.2 months; hazard ratio = 1.00), but increased grade ≥ 3 complications at postoperative day 60 (26% vs. 15%; *p* = 0.035) [3]. Because the trial evaluated a specific oxaliplatin regimen, the role and optimal regimen of HIPEC remain debated [4,5]. For the present study, the relevance of PRODIGE 7 is methodological: regardless of the additional oncologic benefit of HIPEC, CRS with or without HIPEC entails substantial perioperative morbidity that should be measured sensitively for patient counseling, clinical decision-making, and comparisons across centers.

The Clavien–Dindo (CD) classification is the most widely used system for grading the severity of postoperative complications according to the treatment required [6]. Although individual complications can be graded separately, patient-level morbidity is conventionally summarized using only the highest CD grade. This approach does not reflect the additional burden of multiple complications occurring in the same patient. The Comprehensive Complication Index (CCI) was developed to address this limitation by integrating all postoperative complications, weighted according to their severity, into a continuous score ranging from 0 (no complications) to 100 (death) [7].

The CCI has demonstrated greater sensitivity than the conventional highest-grade CD approach for assessing postoperative morbidity in gastrointestinal surgery [8]. Its utility has also been evaluated in patients undergoing CRS-HIPEC. In this setting, the CCI was more strongly associated with length of hospital stay than the highest CD grade [9]. Among patients undergoing CRS-HIPEC for colorectal peritoneal metastases, increasing cumulative complication burden was associated with substantially higher treatment-related healthcare costs [10]. Greater postoperative complication burden has also been associated with poorer cancer-related survival in patients with peritoneal malignancies [11]. Collectively, these findings suggest that cumulative assessment may provide clinically relevant information beyond reporting only the most severe complication.

Despite the increasing use of the CCI, postoperative morbidity after CRS-HIPEC continues to be commonly reported using only the highest CD grade, as illustrated by recent population-based studies [12]. This approach may underrepresent the cumulative burden experienced by patients who develop multiple low- or moderate-grade complications. Direct comparisons of the CCI and the conventional patient-level CD grade in relation to clinically relevant postdischarge outcomes, particularly readmission, remain limited.

Unplanned readmission is a clinically relevant marker of postdischarge recovery because it may reflect late complications, persistent physiological impairment, or an incomplete transition from inpatient to outpatient care, while adding burden for patients and healthcare services. However, readmission is not specific to surgical morbidity and may also be influenced by discharge planning, access to outpatient care, distance from the referral center, and social support. Assessing both 30- and 90-day readmission therefore captures early and later postdischarge events while requiring cautious interpretation of their causes.

The primary aim of this study was to compare the CCI and the conventional patient-level highest CD grade, both derived from postoperative complications occurring during the index hospitalization, in terms of their associations with and discriminative performance for unplanned readmission within 30 and 90 days after discharge in patients undergoing CRS-HIPEC for colorectal peritoneal metastases. Agreement between the dichotomized CCI and CD classifications and the associations of clinical and operative variables with CCI scores were evaluated as secondary exploratory outcomes. Given the limited sample size, all findings were considered hypothesis-generating rather than confirmatory.

## 2. Materials and Methods

### 2.1. Study Design and Patient Population

This was a single-center, retrospective cohort study. Sixty-five consecutive patients who underwent surgical exploration with the intention of performing CRS-HIPEC at our center between May 2018 and December 2025 were screened. Eligible patients were aged >18 years and had a histologically confirmed colorectal primary tumor with synchronous or metachronous peritoneal metastases considered to be of colorectal origin. Both colon and rectal primaries were eligible. Eligibility was not restricted by adenocarcinoma subtype; conventional, mucinous, and signet-ring cell variants were eligible. No patient in the analyzed cohort had extraperitoneal metastatic disease, including controlled or technically resectable disease.

**Figure 1:**
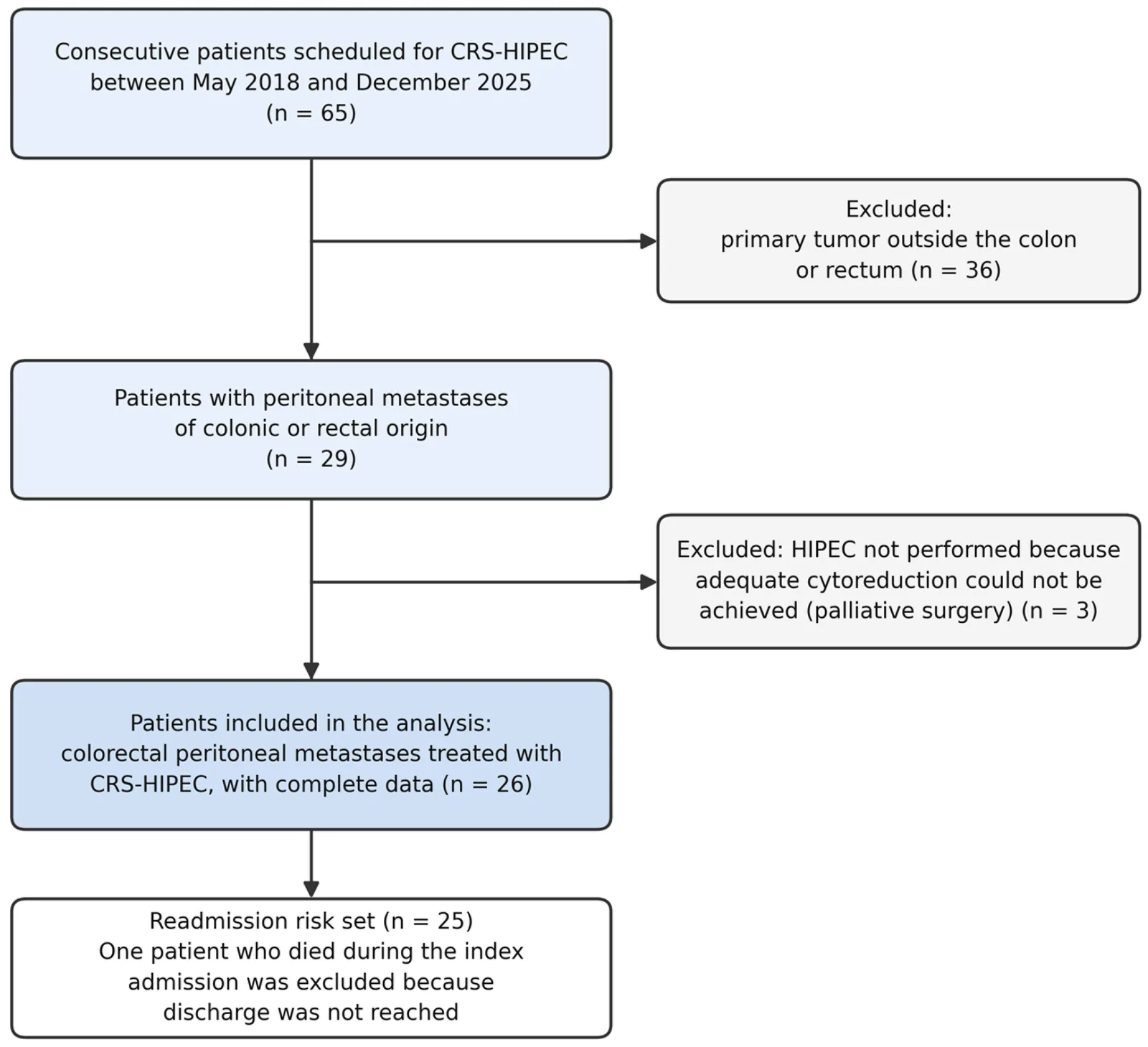
Patient selection flow chart.

Thirty-six patients with a primary tumor originating outside the colon or rectum were excluded. Among the remaining 29 patients with colorectal primary tumors, three did not undergo HIPEC because CC-0 cytoreduction could not be achieved and were therefore excluded. The final cohort comprised 26 patients who underwent CRS-HIPEC for colorectal peritoneal metastases (Figure 1). Each patient contributed one index procedure, and no repeat CRS-HIPEC procedures occurred during the study period. No otherwise eligible patient was excluded because of missing data, loss to follow-up, or inability to ascertain readmission.

The study was conducted in accordance with the Declaration of Helsinki and was approved by the Research Ethics Committee of the Faculty of Medicine, Çukurova University (meeting no. 166, decision no. 49; 8 May 2026). The requirement for individual informed consent was waived because of the retrospective design of the study.

### 2.2. Data Collection

Demographic, clinical, operative, and postoperative data were retrospectively extracted from the hospital information system and patient records. Demographic and clinical variables included age, sex, body mass index, American Society of Anesthesiologists (ASA) class, Eastern Cooperative Oncology Group (ECOG) performance status, Charlson Comorbidity Index, primary tumor location, and receipt of preoperative systemic chemotherapy. Operative variables included the Peritoneal Cancer Index (PCI), completeness of cytoreduction (CC) score, extent of peritonectomy, number of resected organs, stoma formation, creation of an anastomosis, operative time, estimated blood loss, HIPEC agent, and perfusion time. Estimated blood loss represented the total intraoperative volume documented in the anesthesia and operative records. Postoperative data included complications, reoperation, length of hospital stay, in-hospital mortality, and unplanned readmission. Before analysis, the data were checked for completeness and internal consistency, and any discrepancies were verified against the original source records.

### 2.3. Surgical Procedure and HIPEC Protocol

All patients were evaluated by the institutional multidisciplinary tumor board. The indication for CRS-HIPEC was determined on the basis of disease burden, response to systemic treatment, performance status, and the anticipated feasibility of complete macroscopic cytoreduction. Complete macroscopic cytoreduction was defined as a CC-0 score, indicating no visible residual disease at completion of cytoreduction. Cytoreductive surgery was performed using standard surgical principles, with peritonectomy procedures and visceral resections undertaken as required to achieve CC-0 cytoreduction. The extent of peritonectomy was determined intraoperatively according to the distribution of peritoneal disease. The PCI was assessed at surgical exploration before cytoreduction, and the completeness of cytoreduction (CC) score was assigned after completion of the cytoreductive procedure. HIPEC was performed only after CC-0 cytoreduction. Patients in whom complete macroscopic cytoreduction could not be achieved underwent palliative surgery without HIPEC and were excluded from the analyzed cohort.

HIPEC was performed using the closed-abdomen technique, with the intraperitoneal temperature maintained at 42–43 °C. Twenty-five patients (96.2%) received intraperitoneal oxaliplatin 460 mg/m^2^ with concomitant intravenous 5-fluorouracil 400 mg/m^2^ and leucovorin 20 mg/m^2^; one patient received intraperitoneal cisplatin 50 mg/m^2^. The cisplatin perfusion lasted 60 min. The median perfusion time for the cohort was 52.5 min (IQR 45.0–60.0; range 30–90 min); the single 90-min perfusion used oxaliplatin. The closed technique, target temperature, and agent-specific doses remained essentially unchanged during the 2018–2025 study period.

### 2.4. Classification of Complications: CCI and the Conventional Clavien–Dindo Classification

Two general surgery specialists, one of whom was an associate professor, independently reviewed the complete medical records to identify all postoperative complications occurring between CRS-HIPEC and discharge or in-hospital death, including lower-grade events; they did not grade only a precompiled complication list. Each identified event was independently graded according to the Clavien–Dindo classification [6], after which the CCI was calculated from the finalized set of events using the standard formula described by Slankamenac et al. [7]. For patients with multiple complications, the conventional patient-level CD grade was defined by the highest recorded grade, whereas all complication events were included in the CCI calculation. Postoperative blood transfusions were classified as grade II, and reoperations under general anesthesia as at least grade IIIb [6]. The reviewers applied the original CD criteria and the prespecified classification rules described above. They were not blinded to readmission status because this information was available in the same records. The number of initial disagreements was not retained; disagreements were resolved by discussion and consensus between the two reviewers.

A CCI threshold of ≥26.2, corresponding to a single CD grade IIIa complication, was used to define a high cumulative complication burden. Major morbidity according to the conventional CD classification was defined as grade ≥ IIIa. This threshold was used as a clinically interpretable anchor rather than as a universally validated cut-off. Because every patient with a CD grade ≥ IIIa necessarily had a CCI ≥ 26.2, discordance could occur only in patients with a CD grade < IIIa but a CCI ≥ 26.2.

### 2.5. Outcomes and Other Definitions

Length of hospital stay was calculated from the date of CRS-HIPEC to discharge or in-hospital death. Unplanned readmission was defined as any unplanned rehospitalization for any cause within 30 or 90 days after discharge from the CRS-HIPEC admission. The 90-day outcome was cumulative and therefore included readmissions occurring during the first 30 days. For each binary outcome, patients were counted once regardless of the number of readmission episodes within the relevant time window. Patients who died before discharge were excluded from the readmission analyses because they did not enter the postdischarge risk period.

Readmissions were identified using both our center’s hospital information system and the national electronic health record system (e-Nabız), allowing admissions to other hospitals to be captured. At least 90 days of follow-up information was available for all discharged patients. Thirty- and 90-day unplanned readmissions were treated as co-primary outcomes. No adjustment for multiplicity was applied; therefore, all analyses were considered exploratory. Agreement between the CCI and CD classifications and the associations of clinical and operative variables with CCI scores were evaluated as secondary exploratory outcomes.

### 2.6. Statistical Analysis

Continuous variables were summarized as median and interquartile range (IQR), and categorical variables as number and percentage. Continuous variables were compared using the Mann–Whitney U test, and categorical variables using Fisher’s exact test. Associations of the dichotomized CCI and CD classifications with readmission were summarized using conditional maximum-likelihood odds ratios (ORs) with exact conditional 95% confidence intervals (CIs).

Agreement between the dichotomized classifications (CCI ≥ 26.2 and CD ≥ IIIa) was assessed using Cohen’s kappa coefficient with a 95% CI. The exact McNemar test was used to evaluate whether discordance between the paired classifications was asymmetric. The discriminative performance of the continuous CCI and ordinal CD grade for readmission was evaluated using the area under the receiver operating characteristic curve (AUC). The CD grade was treated as an ordinal variable ranging from 0 (no complication) to 7 (grade V), with grades IIIa/IIIb and IVa/IVb represented as separate ordered categories. AUC 95% CIs were obtained using 10,000 outcome-stratified bootstrap replicates. Differences between the two AUCs were evaluated using paired bootstrap CIs and the DeLong test for correlated ROC curves [13,14]. Given the small risk set, DeLong *p* values were considered approximate.

Associations of clinical and operative variables with CCI scores were evaluated using Spearman’s rank correlation. These analyses were repeated after excluding the patient who died during the index hospitalization and had a CCI of 100. All tests were two-sided, and *p* < 0.05 was considered statistically significant. Analyses were performed using Python version 3.11.15 (NumPy 2.4.4, SciPy 1.17.1, and pandas 3.0.2). The analysis script is available upon reasonable request.

## 3. Results

### 3.1. Patient and Treatment Characteristics

The study included 26 patients who underwent CRS-HIPEC for colorectal peritoneal metastases. The median age was 60.5 years (IQR 43.2–69.8), and 14 patients (53.8%) were male. The primary tumor was located in the colon in 19 patients (73.1%) and in the rectum in 7 (26.9%). The median PCI was 8.0 (IQR 5.0–12.8), and CC-0 cytoreduction was achieved in all patients. Twenty-one patients (80.8%) had received preoperative systemic chemotherapy. Additional demographic, clinical, and operative characteristics are presented in Table 1.

### 3.2. Assessment of Complications with the CCI and the Conventional Clavien–Dindo Classification

A total of 63 postoperative complication events were recorded in 25 of the 26 patients (96.2%). The mean number of events was 2.4 per patient (median 2; range 0–6), and 20 patients (76.9%) experienced at least two complications. The median CCI was 33.5 (IQR 24.4–39.5), and the mean was 35.4 ± 18.1. Major morbidity according to the conventional CD classification (grade ≥ IIIa) was present in 13 patients (50.0%), whereas 19 patients (73.1%) had a high cumulative complication burden according to the CCI (≥26.2). The two classifications agreed in 20 patients (76.9%), with moderate agreement (kappa = 0.54; 95% CI 0.25–0.83). Six patients (23.1%) had a CD grade < IIIa but a CCI ≥ 26.2, whereas no discordance occurred in the opposite direction (exact McNemar *p* = 0.031). The distributions of complications and patient-level cumulative burden are provided in Supplementary Tables S1 and S2, respectively, and the principal postoperative outcomes are summarized in Table 2.

### 3.3. Main Analysis: Association of the CCI and the Conventional Clavien–Dindo Classification with Readmission

Among the 25 discharged patients included in the readmission analyses, 8 (32.0%) were readmitted within 30 days and 11 (44.0%) within 90 days. The 30-day readmission rate was 38.9% (7/18) in patients with a CCI ≥ 26.2 and 14.3% (1/7) in those with a CCI < 26.2 (OR = 3.64; 95% CI 0.32–200.29; *p* = 0.362). The corresponding 90-day rates were 55.6% (10/18) and 14.3% (1/7), respectively (OR = 6.95; 95% CI 0.64–378.69; *p* = 0.090). According to the conventional CD classification, 30-day readmission occurred in 33.3% (4/12) of patients with grade ≥ IIIa and 30.8% (4/13) of those with grade < IIIa (OR = 1.12; 95% CI 0.15–8.32; *p* = 1.000). The corresponding 90-day rates were 50.0% (6/12) and 38.5% (5/13), respectively (OR = 1.57; 95% CI 0.25–10.39; *p* = 0.695). Although the readmission rates were numerically higher in the CCI ≥ 26.2 group, none of these associations reached statistical significance, and all confidence intervals were wide (Table 3).

Eight patients were readmitted within 30 days after discharge. The recorded reasons for readmission were elevated creatinine levels (n = 1), intra-abdominal collection managed nonoperatively (n = 2), elevated liver enzyme levels (n = 1), deterioration in general condition (n = 1), surgical-site infection (n = 2), and abdominal pain (n = 1). Three additional patients were first readmitted between days 31 and 90 because of ileus (n = 2) or inadequate oral intake (n = 1). Overall, 11 patients were readmitted within 90 days. Six of these 11 patients were readmitted for clearly defined surgical complications: two intra-abdominal collections, two surgical-site infections, and two cases of ileus. For the remaining five patients, a direct causal relationship between readmission and a specific postoperative surgical complication could not be established reliably from the retrospective records.

When analyzed as a continuous variable, the median CCI was 34.1 (IQR 32.5–36.2) in patients readmitted within 30 days and 29.6 (IQR 20.9–44.7) in those who were not readmitted (*p* = 0.703). For 90-day readmission, the corresponding median CCI values were 34.6 (IQR 31.5–37.9) and 29.6 (IQR 20.9–38.0), respectively (*p* = 0.151). Neither difference was statistically significant (Table 4).

For 30-day readmission, the AUC was 0.551 (95% CI 0.331–0.779) for the CCI and 0.485 (95% CI 0.283–0.691) for the ordinal CD grade. The AUC difference was 0.066 (paired bootstrap 95% CI −0.077 to 0.232; DeLong *p* = 0.419). For 90-day readmission, the AUC was 0.672 (95% CI 0.445–0.870) for the CCI and 0.555 (95% CI 0.344–0.760) for the CD grade. The AUC difference was 0.117 (paired bootstrap 95% CI −0.006 to 0.253; DeLong *p* = 0.074). Although the CCI showed numerically higher discrimination for 90-day readmission, the difference between the two measures was not statistically significant at either time point (Table 5).

### 3.4. Parameters Associated with the Level of the CCI

Associations between the continuous CCI score and clinical and operative variables are presented in Table 6. Reoperation was not evaluated as a correlate because it is itself a component of the CCI.

The CCI showed positive correlations with body mass index (BMI) (rho = 0.541; *p* = 0.004) and the Peritoneal Cancer Index (PCI) (rho = 0.423; *p* = 0.031). No statistically significant correlations were observed with age, ECOG performance status, ASA class, Charlson Comorbidity Index, operative time, or estimated blood loss. CCI scores also did not differ significantly according to the extent of peritonectomy (total vs. partial: median 34.1 vs. 31.5; *p* = 0.371). After exclusion of the patient who died during the index hospitalization and had a CCI of 100, the correlations with BMI (rho = 0.497; *p* = 0.012) and PCI (rho = 0.458; *p* = 0.021) remained. These analyses were exploratory and were not adjusted for multiple comparisons. Because of the limited sample size, no multivariable analysis was performed; therefore, the association with PCI should be interpreted cautiously.

### 3.5. Exploratory Analysis: Subgroups According to Classification Agreement

Discordance between the CD and CCI classifications was observed in 6 patients (23.1%). All six had a highest CD grade of II but a CCI ≥ 26.2 because they experienced two or three grade II complications during the same hospitalization. Postoperative blood transfusion was recorded in all six patients, together with one or more additional grade II events, as detailed in Supplementary Table S3. Based on classification agreement, the cohort was divided into three subgroups: concordant without major morbidity (CD < IIIa and CCI < 26.2), discordant (CD < IIIa and CCI ≥ 26.2), and concordant with major morbidity (CD ≥ IIIa and CCI ≥ 26.2). Given the small subgroup sizes, these comparisons were considered descriptive (Table 7).

The median length of hospital stay was 7.0 days in the concordant group without major morbidity, 11.5 days in the discordant group, and 21.0 days in the concordant group with major morbidity. In the discordant group, the 30- and 90-day readmission rates were 50.0% and 66.7%, respectively, compared with 14.3% for both outcomes in the concordant group without major morbidity and 33.3% and 50.0%, respectively, in the concordant group with major morbidity. These findings were descriptive, and no formal statistical comparisons were performed because of the small subgroup sizes.

## 4. Discussion

This retrospective cohort study of 26 patients undergoing CRS-HIPEC for colorectal peritoneal metastases found moderate agreement between the conventional CD classification and the CCI. Six patients classified as having no major morbidity by the CD classification had a high cumulative burden according to the CCI. Patients with CCI ≥ 26.2 had numerically higher 30- and 90-day readmission rates, and the CCI had a numerically higher AUC than the ordinal CD grade for 90-day readmission. However, neither the associations with readmission nor the differences in discrimination were statistically significant. CCI scores were also positively correlated with BMI and PCI in exploratory univariable analyses. The findings support the descriptive value of cumulative morbidity assessment but do not establish superiority of the CCI for readmission.

The 23.1% discordance rate reflects the different structures of the two instruments. The conventional patient-level CD approach retains only the most severe event, whereas the CCI accumulates all events. Because a CCI of 26.2 corresponds to one grade IIIa complication, every patient with CD grade ≥ IIIa necessarily met the CCI threshold; discordance could occur only when multiple lower-grade complications accumulated above that threshold. All six discordant patients had two or three grade II complications, and all had a postoperative transfusion. Excluding transfusion would have moved four of the six below the CCI threshold, meaning that two-thirds of the observed discordance depended on counting this event. Institutional transfusion thresholds and documentation practices may therefore materially influence the frequency of CCI–CD discordance and limit its generalizability across centers. The significant McNemar result demonstrates asymmetric classification but not clinical superiority. In practice, the CCI may complement rather than replace the widely used CD classification when complication ascertainment is sufficiently standardized.

Although higher CCI values were consistently associated with numerically higher readmission rates in our study, these associations did not reach statistical significance. The small risk set (n = 25, with 8 and 11 outcome events at 30 and 90 days, respectively) and the resulting wide confidence intervals—for example, 0.64–378.69 for the 90-day OR comparing the dichotomized CCI groups and −0.006 to 0.253 for the difference in 90-day AUCs—indicate considerable statistical uncertainty. Evidence from larger cohorts nevertheless supports the clinical relevance of postoperative morbidity. Choudry et al. [11], in a cohort of 1296 patients with peritoneal malignancies, reported that postoperative complications independently predicted both 30-day readmission and cancer-related survival. Similarly, Ortiz-López et al. [15] found that the CCI was independently associated with overall survival among 604 patients undergoing colorectal cancer surgery. Although these studies evaluated different patient populations and outcomes and therefore do not directly validate our readmission findings, they support further investigation of cumulative postoperative morbidity as a potentially relevant clinical measure. With the available data, we cannot determine whether the observed numerical trend reflects a true association or sampling variation.

Factors associated with the CCI have varied across studies, possibly because of differences in patient populations, measured covariates, and analytical methods. Eng et al. [16] found that PCI was associated with the CCI in univariable analysis among patients undergoing CRS-HIPEC, whereas the change in base excess at 48 h after surgery was the only independent predictor in their multivariable model. The direction of the univariable association between PCI and the CCI is consistent with our finding (rho = 0.423; *p* = 0.031) and is clinically plausible because PCI reflects the extent of peritoneal disease and may be associated with the complexity of cytoreduction. In a larger cohort undergoing CRS with or without HIPEC, Tejedor et al. [17] identified pre-existing cardiac disease, the number of anastomoses, and fluid balance during the first 24 h as independent risk factors for CCI-defined major morbidity, whereas higher preoperative hemoglobin and opioid-free anesthesia were protective factors. Several of these variables were not available in our dataset, which may partly explain the differences between the studies. The association observed between BMI and the CCI in our cohort (rho = 0.541; *p* = 0.004) may reflect the relationship between body composition, technical difficulty, and postoperative morbidity; however, given the limited BMI distribution (median 23.7 kg/m^2^) and small sample size, this finding should be regarded as exploratory. Moreover, all associations in our study were evaluated using univariable analyses without adjustment for multiple comparisons.

The rate of major complications according to the conventional CD classification (≥IIIa) in our cohort was 50.0%, placing it at the upper end of the rates reported in the literature. Tejedor et al. [17] reported a major morbidity rate of 27.4%, defined using a study-specific CCI threshold of ≥22.6 corresponding to the upper quartile of their cohort; this differs from the clinically anchored threshold of CCI ≥ 26.2 used in our study. Rintala et al. [18] reported a 30-day CD grade III–V complication rate of 40% among 171 patients undergoing CRS, whereas the PRODIGE 7 trial reported grade ≥ 3 adverse events within 30 days in 42% of patients in the CRS-HIPEC group [3]. These rates are not directly comparable because of differences in complication definitions, grading systems, assessment periods, and patient populations. The relatively high major morbidity rate in our cohort may also reflect the extensive procedures performed in some patients to achieve complete cytoreduction, although this cannot be directly established from the available data.

The 30- and 90-day readmission rates in our cohort were 32.0% and 44.0%, respectively, and were higher than those reported in the limited number of comparable studies. Kazanowski et al. [19] reported a 30-day readmission rate of 1.9% following CRS-HIPEC, whereas Rintala et al. [18] reported a readmission rate of 13% in the colorectal peritoneal metastasis subgroup undergoing CRS (n = 73), although the corresponding time window was not specified. Six of our 11 readmissions were for clearly defined surgical complications—two intra-abdominal collections, two surgical-site infections, and two ileus episodes. The remaining indications were medical or nonspecific, and their causal relationship to the index procedure could not be determined reliably from retrospective records. These findings illustrate that all-cause readmission is clinically informative but broader than surgical morbidity alone. Differences between studies may reflect the definition and starting point of readmission, duration of follow-up, discharge and outpatient-care practices, referral distance, social support, center-specific presentation patterns, and the large effect of individual events in a small cohort. The heterogeneity of readmission definitions in the CRS-HIPEC literature limits direct comparisons and highlights the need for standardized reporting.

Although these findings require confirmation, they suggest two potential implications for further clinical investigation. First, reporting postoperative morbidity solely according to the conventional CD classification may underestimate the cumulative burden in patients who experience multiple moderate complications during the same admission. When complications are recorded systematically, calculating the CCI alongside—rather than instead of—the CD classification may provide complementary information for institutional quality assessment and, with standardized event ascertainment, comparisons between centers. Second, whether patients classified as having no major complication by the CD classification but a high cumulative burden according to the CCI might benefit from closer post-discharge follow-up warrants evaluation in larger prospective cohorts. The present study also has several methodological strengths. Individual complications were graded independently by two investigators using an explicit prespecified rule, including the classification of reoperation under general anesthesia as at least grade IIIb. The 30- and 90-day readmission windows, their calculation from the date of discharge, and the cumulative definition of 90-day readmission were specified consistently and verified at the patient level. In addition, the discriminative performances of the CCI and the conventional CD classification were directly compared using bootstrap confidence intervals and the DeLong test. Finally, restricting the cohort to peritoneal metastases of colonic and rectal origin reduced the biological heterogeneity that might have resulted from including appendiceal malignancies.

The limitations fall into four domains. First, the retrospective, single-center design restricts external validity. Because the analysis included only patients who achieved CC-0 cytoreduction and completed HIPEC, the findings do not apply to patients with incomplete cytoreduction or exploration without HIPEC.

Second, the readmission analysis included only 25 discharged patients, with 8 events by 30 days and 11 by 90 days. The resulting confidence intervals were very wide; the absence of statistically significant differences should therefore not be interpreted as evidence of equivalence between the CCI and the conventional CD classification.

Third, exposure and outcome measurement depended on retrospective records. Complications were limited to the index hospitalization, so low-grade or incompletely documented events may have been missed and postdischarge complications did not contribute to the CCI. The reviewers were not blinded to readmission status, and the number of initial classification disagreements was not recorded. Both classifications depended on the same source records. Transfusion was present in all six discordant cases, so the observed discordance may vary with institutional transfusion and documentation practices. In addition, all-cause readmission may be affected by discharge planning, outpatient access, referral distance, and social circumstances beyond index-admission morbidity.

Fourth, statistical inference was exploratory. Odds ratios were unadjusted, CCI-associated factors were examined only in univariable analyses, and no correction was made for multiple comparisons. The CCI ≥ 26.2 threshold was a clinically interpretable anchor rather than a validated prognostic cut-off for readmission, and its structural relationship with CD grade ≥ IIIa limits interpretation of the McNemar comparison. The study therefore provides no prognostic validation, and its findings require confirmation in larger, prospective, multicenter cohorts.

## 5. Conclusions

In this cohort, the CCI identified a high cumulative complication burden in 6 of 26 patients (23.1%) who were classified as having no major complication by the conventional CD classification. Although readmission rates were numerically higher among patients with a high CCI and the CCI showed a higher AUC than the conventional CD classification for 90-day readmission, neither the association with readmission nor the difference between the two classification systems reached statistical significance. These findings suggest that the CCI may complement the conventional CD classification by capturing the cumulative burden of multiple complications; however, its additional value for predicting readmission after CRS-HIPEC remains unproven. Larger, prospective, multicenter studies are required to determine whether the CCI provides clinically meaningful improvement in postoperative risk stratification.

## Figures and Tables

**Table 1:** Demographic, clinical and operative characteristics of the cohort (*n* = 26).

Variable	Value (n = 26)
Age (years), median (IQR)	60.5 (43.2–69.8)
Sex, male/female, n	14/12
Body mass index (BMI) (kg/m^2^), median (IQR)	23.7 (22.0–26.5)
ASA class, n (%), I/II/III	1 (3.8)/17 (65.4)/8 (30.8)
ECOG performance score, n (%), 0/1/2/3	14 (53.8)/10 (38.5)/1 (3.8)/1 (3.8)
Charlson Comorbidity Index, median (IQR)	3.0 (2.0–5.8)
Primary tumor location, colon/rectum ^†^, n (%)	19 (73.1)/7 (26.9)
Peritoneal Cancer Index (PCI), median (IQR)	8.0 (5.0–12.8)
Completeness of cytoreduction (CC score), CC-0, n (%)	26 (100.0)
Extent of peritonectomy, total/partial, n (%)	8 (30.8)/18 (69.2)
Number of organs resected, median (IQR)	2.0 (1.3–3.0)
Stoma formation, n (%)	14 (53.8)
Anastomosis performed, n (%)	15 (57.7)
Operative time (min), median (IQR)	360 (287.5–405.0)
Estimated blood loss (mL), median (IQR)	100 (100–200)
HIPEC agent, oxaliplatin-based, n (%) ^‡^	25 (96.2)
HIPEC perfusion time (min), median (IQR)	52.5 (45.0–60.0)
Preoperative systemic chemotherapy, n (%)	21 (80.8)

IQR: interquartile range (25th–75th percentile). ASA: American Society of Anesthesiologists; ECOG: Eastern Cooperative Oncology Group. ^†^ One patient with a rectosigmoid location was included in the rectum group. ^‡^ Cisplatin was used in the remaining patient.

**Table 2:** Classification of postoperative complications by the conventional Clavien–Dindo classification and the CCI, and short-term outcomes (*n* = 26).

Variable	Value (n = 26)
Conventional Clavien–Dindo grade, *n* (%)	
No complication	1 (3.8)
Grade I	0 (0.0)
Grade II	12 (46.2)
Grade IIIa	9 (34.6)
Grade IIIb	3 (11.5)
Grade V (death)	1 (3.8)
Major complication by conventional CD (≥IIIa), *n* (%)	13 (50.0)
High cumulative complication burden by CCI (≥26.2), *n* (%)	19 (73.1)
Overall agreement between the two classifications, *n* (%)	20/26 (76.9)
Agreement between the two classifications (Cohen’s kappa, 95% CI)	0.54 (0.25–0.83); moderate
Paired comparison of the two classifications (exact McNemar *p*)	0.031
Length of stay (days), median (IQR)	11.5 (8.0–20.5)
30-day readmission, *n* (%) ^†^	8/25 (32.0)
90-day readmission, *n* (%) ^†^	11/25 (44.0)
Reoperation, *n* (%)	2 (7.7)
In-hospital mortality, *n* (%)	1 (3.8)

^†^ Readmission was defined from the date of discharge; the 1 patient who died during the index admission was removed from both the 30- and the 90-day risk set because discharge was not reached (n = 25). The 90-day definition was pre-specified as a cumulative window that also covers the first 30 days.

**Table 3:** Thirty- and 90-day readmission according to the dichotomized CCI and conventional Clavien–Dindo groups.

Classification	30-Day Readmission, n/N (%)	OR (95% CI) *	*p* ^†^	90-Day Readmission, n/N (%)	OR (95% CI) *	*p* ^†^
CCI < 26.2 (reference)	1/7 (14.3)	1	–	1/7 (14.3)	1	–
CCI ≥ 26.2	7/18 (38.9)	3.64 (0.32–200.29)	0.362	10/18 (55.6)	6.95 (0.64–378.69)	0.090
CD < IIIa (reference)	4/13 (30.8)	1	–	5/13 (38.5)	1	–
CD ≥ IIIa	4/12 (33.3)	1.12 (0.15–8.32)	1.000	6/12 (50.0)	1.57 (0.25–10.39)	0.695

* Unadjusted conditional maximum-likelihood odds ratio with an exact conditional 95% confidence interval. ^†^ Fisher’s exact test. Both analyses included 25 discharged patients; the patient who died before discharge was excluded.

**Table 4:** Comparison of CCI scores according to readmission status.

Outcome	CCI in Readmitted Patients, Median (IQR)	CCI in Non-Readmitted Patients, Median (IQR)	Mann–Whitney *p*
30-day readmission (*n* = 25)	34.1 (32.5–36.2)	29.6 (20.9–44.7)	0.703
90-day readmission (*n* = 25)	34.6 (31.5–37.9)	29.6 (20.9–38.0)	0.151

IQR: interquartile range. The 1 patient who died during the index admission is excluded from both risk sets.

**Table 5:** Comparison of the discriminative performance of the CCI and of the conventional Clavien–Dindo grade (ordinal, 0–7) for readmission by ROC-AUC.

Outcome	CCI AUC (95% CI)	CD AUC (95% CI)	AUC Difference (95% CI) *	DeLong *p*
30-day readmission (*n* = 25)	0.551 (0.331–0.779)	0.485 (0.283–0.691)	0.066 (−0.077 to 0.232)	0.419
90-day readmission (*n* = 25)	0.672 (0.445–0.870)	0.555 (0.344–0.760)	0.117 (−0.006 to 0.253)	0.074

The AUC was calculated from the Mann–Whitney U statistic. * The 95% confidence intervals for each AUC and for the AUC difference were obtained by the percentile method from 10,000 outcome-stratified bootstrap replicates, the difference being resampled as a paired quantity within the same replicates. The DeLong *p* value refers to the comparison of the two dependent ROC curves.

**Table 6:** Univariable Spearman correlations between the level of the CCI and clinical and operative parameters.

Variable	Spearman rho	*p* Value
Body mass index (BMI)	0.541	0.004
Peritoneal Cancer Index (PCI)	0.423	0.031
Age	0.329	0.101
ECOG performance score	0.328	0.102
ASA class	0.224	0.272
Estimated blood loss	0.181	0.375
Operative time	0.159	0.438
Charlson Comorbidity Index	−0.047	0.821

**Table 7:** Exploratory comparison of the three subgroups defined by classification agreement status.

Group	n	Length of Stay (Days), Median (IQR)	30-Day Readmission, n/N (%)	90-Day Readmission, n/N (%)
Concordant, no major complication (CD < IIIa, CCI < 26.2)	7	7.0 (7.0–9.0)	1/7 (14.3)	1/7 (14.3)
Discordant (CD < IIIa, CCI ≥ 26.2)	6	11.5 (9.2–13.0)	3/6 (50.0)	4/6 (66.7)
Concordant, major complication (CD ≥ IIIa, CCI ≥ 26.2)	13 *	21.0 (15.0–26.0)	4/12 (33.3)	6/12 (50.0)

* Length of stay was calculated for all 13 patients; readmission rates were calculated for n = 12 because the 1 patient who died during the index admission was removed from the risk set (see the footnote to Table 3).

## Data Availability

The data presented in this study are available on request from the corresponding author. The data are not publicly available owing to privacy restrictions. The analysis script is available upon request.

## Supplementary Materials

The following supporting information can be downloaded at: https://www.mdpi.com/article/10.3390/medicina62101935/s1, Table S1: Postoperative complications of the index admission by type and Clavien–Dindo grade; Table S2: Burden of complications per patient; Table S3: Complications of the six patients classified as having no major complication by the Clavien–Dindo classification but a high cumulative burden by the CCI.

## Abbreviations

The following abbreviations are used in this manuscript:

ASA	American Society of Anesthesiologists
AUC	Area under the curve
BMI	Body mass index
CC	Completeness of cytoreduction
CCI	Comprehensive Complication Index
CD	Clavien–Dindo
CI	Confidence interval
CRS	Cytoreductive surgery
CRS-HIPEC	Cytoreductive surgery with hyperthermic intraperitoneal chemotherapy
ECOG	Eastern Cooperative Oncology Group
HIPEC	Hyperthermic intraperitoneal chemotherapy
IQR	Interquartile range
OR	Odds ratio
PCI	Peritoneal Cancer Index
ROC	Receiver operating characteristic
