# Supplementary material for: Comprehensive Complication Index Versus Clavien–Dindo Classification After CRS-HIPEC for Colorectal Peritoneal Metastases: Associations with 30- and 90-Day Readmission
Source: Medicina (Kaunas). 2026 Oct 7;62(10):1935. doi: 10.3390/medicina62101935 (PMC13707154; doi:10.3390/medicina62101935)
Supplement: Supplementary file 1 [file medicina-62-01935-s001.zip › medicina-4585727-supplementary.pdf]

Table S1. Postoperative complications of the index admission by type and Clavien–Dindo grade.

Complication	Grade I	Grade II	Grade IIIa	Grade IIIb	Grade V
Postoperative blood transfusion	–	22	–	–	–
Fever	2	8	–	–	–
Surgical site infection	–	6	4	–	–
Medical (Acute kidney injury, myocardial infarction, hyponatremia, hyperglycemia, hypertension)	–	6	1	–	–
Pleural effusion	–	–	6	–	–
Intra-abdominal collection	–	2	–	1	–
Postoperative bleeding requiring reoperation	–	–	–	1	–
Pneumonia	–	1	–	–	–
Ileus	–	1	–	–	–
Anastomotic leak	–	–	–	1	–
Sepsis	–	–	–	–	1
Total events (n = 63)	2	46	11	3	1

Table S2. Burden of complications per patient.

	n	%
Patients with at least one complication	25	96.2
Patients with no complication	1	3.8
Patients with ≥2 complications	20	76.9
Patients with ≥3 complications	11	42.3
Total complication events	63	–
Complications per patient, mean (median, range)	2.4 (2.0, 0–6)	–

Table S3. Complications of the six patients classified as having no major complication by the Clavien–Dindo classification but a high cumulative burden by the CCI.

Patient	Complications of the index admission (all Clavien–Dindo grade II)	Events, n	CCI	Highest CD grade
D1	Blood transfusion; fever; surgical site infection	3	36.2	II
D2	Blood transfusion; fever; ileus	3	36.2	II
D3	Blood transfusion; surgical site infection	2	29.6	II
D4	Blood transfusion; surgical site infection	2	29.6	II
D5	Blood transfusion; acute kidney injury	2	29.6	II
D6	Blood transfusion; hyponatremia	2	29.6	II
